# Integration: Gospel for immune bioinformatician on epitope-based therapy

**DOI:** 10.3389/fimmu.2023.1075419

**Published:** 2023-01-31

**Authors:** Baozeng Sun, Junqi Zhang, Zhikui Li, Mingyang Xie, Cheng Luo, Yongkai Wang, Longyu Chen, Yueyue Wang, Dongbo Jiang, Kun Yang

**Affiliations:** ^1^ Department of Immunology, Basic Medicine School, Air-Force Medical University (the Fourth Military Medical University), Xi’an, Shaanxi, China; ^2^ The Key Laboratory of Bio-hazard Damage and Prevention Medicine, Basic Medicine School, Air-Force Medical University (the Fourth Military Medical University), Xi’an, Shaanxi, China; ^3^ Department of Microbiology, Basic Medicine School, Air-Force Medical University (the Fourth Military Medical University), Xi’an, Shaanxi, China; ^4^ Department of Rheumatology, Tangdu Hospital, Air-Force Medical University (the Fourth Military Medical University), Xi’an, Shaanxi, China

**Keywords:** integration, epitope, immunotherapy, *in silico*, bioinformatics, immune response

## 1 Introduction

Peptide-based vaccines are attracting considerable attention due to the potential to precisely tune the immune response using antigens fragments or peptides, as well as the relative ease of production (1). So far, peptide vaccines against viral infectious diseases have been widely developed and entered phase I/II clinical trials (2), which include COVID-19, HIV, influenza, hepatitis B, and hepatitis C, meaning the mature technology in research and development. Also, NeuVax, a peptide vaccine against breast cancer, completed phase III clinical trial and declared safety *in vivo (* 3). This is the furthest step in the quest for epitope vaccines for use in humans to date (4, 5), suggesting the effectiveness of strategies and the bright application prospect to design peptide-based vaccines (6, 7).

Effective epitopes play a major therapeutic role in synthetic peptide vaccines. Identifying and screening epitopes, however, is usually an endless and complex process (8). Fortunately, researchers have developed *in silico* prediction methods that dramatically reduce the burden associated with epitope mapping by minimizing the list of potential epitope candidates for experimental testing (9). These tools allow investigators to analyze antigenic properties at multiple levels and multiple dimensions, such as affinity, immunogenicity, toxicity, and sensitization, which greatly promotes the further comprehension of pathogens and has a great role in promoting the research and development of drugs to benefit disease prevention and control (9–11).

However, the accuracy of single prediction analysis is often only 50%-70% (12–16). This is probably because of the inherent defects in computer algorithms based on epitope databases for fitting functions (8, 11). In view of above deficiencies, we propose a novel train of thought, “integration”, to augment the breadth and depth of bioinformatics in peptide-based therapeutics. At the same time, we call on researchers to excavate connotations with the characteristics of antigens behind the credit tool and to make breakthrough in the prevention and control of diseases such as infection and cancer.

**Graphical Abstract:**
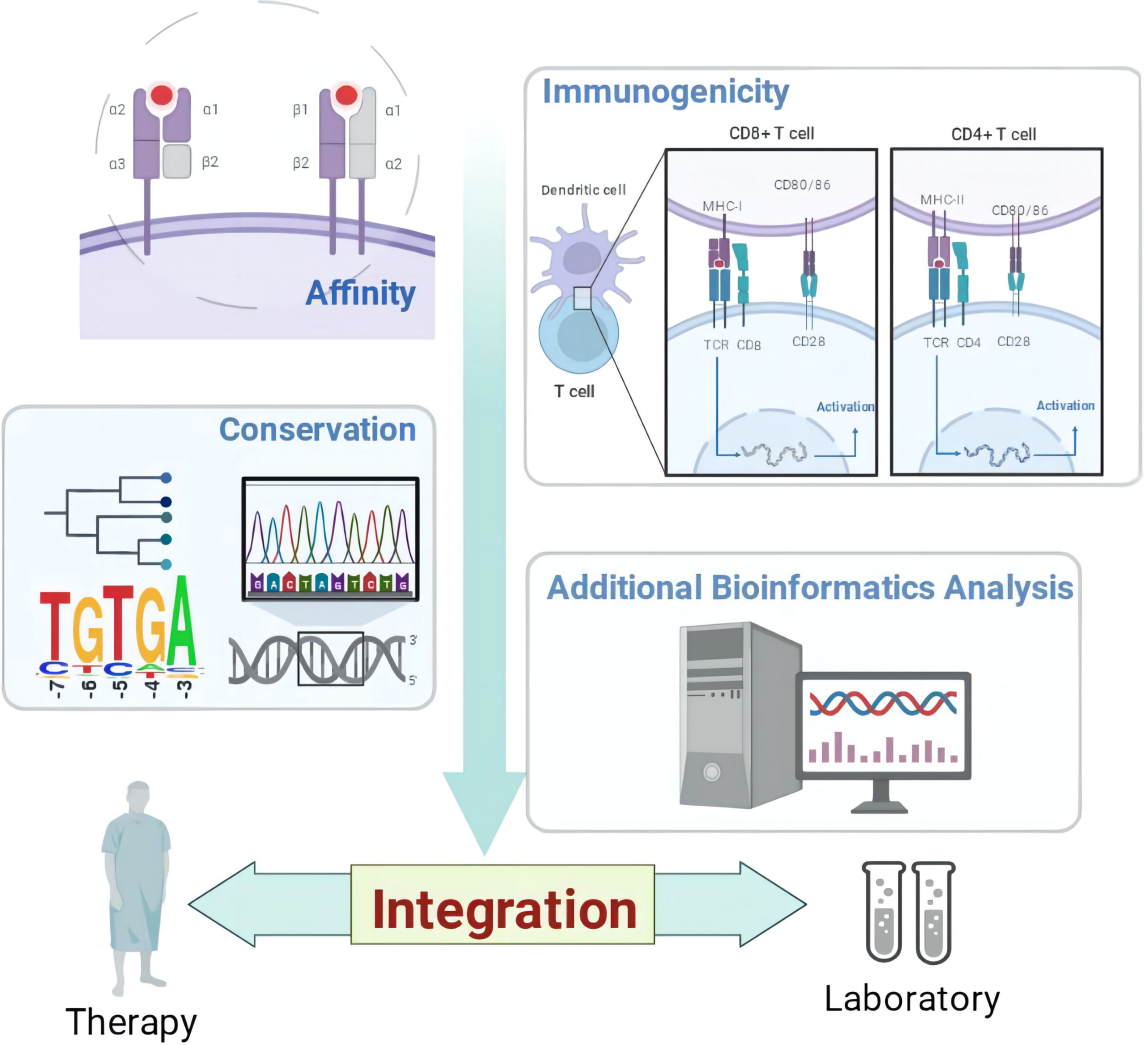


### 1.1 “Integration”: Improving the accuracy of the analysis

Accuracy has always been the goal pursued by various bioinformatics algorithms. Therefore, researchers have relied on a variety of algorithms, such as artificial neural networks (ANNs) (17–19), the stabilized matrix method (SMM) (20), and the consensus method (21), and tested them in tens of thousands of epitopes in multiple databases (17, 18). However, fully accurate algorithmic toolkits have not been developed to date. Meanwhile, *in silico* studies usually reveal their results with limited or even single predictive tools in respective orientations, which greatly hinders the application in general. Therefore, the concept of “integration” is advocated, which is the use of multiple analysis algorithms for an identical antigen, selecting the dominant epitopes predicted by each tool or adopting higher criteria. Considering this strategy in a purpose-oriented way, alternative methods output epitopes by different algorithms, collateral with false positives due to inherent defects of each toolkit. Theoretically, dominant epitopes should be defined through the intersection of multiple tools, which greatly reduces the probability of nondominant epitope output, thereby improving the accuracy of prediction.

Affinity analyses have been widely used in the study of infectious diseases (22–24), neoplasms (25) and autoimmune diseases (26). **Table 1** lists the current international mainstream epitope analysis algorithms. There are multiple algorithms and platforms to analyze affinity, and the IEDB analysis resource (MHC-I binding and MHC-II binding) and DTU HEALTH TECH (NetMHCpan and NetMHCIIpan) platforms have taken over the absolute principal position. The functionality of affinity analysis provided by the IEDB Analytical Resources integrates multiple analytical methods (27), including Artificial neural network (ANN) (19), Stabilized matrix method (SMM) (20), SMM with a Peptide: major histocompatibility complex(MHC) Binding Energy Covariance matrix (SMMPMBEC) (28), and so on. Why not make full use of these algorithms to sharpen the accuracy of affinity analysis? Recently, immunologists followed this thought in glycoprotein epitope studies of Hantaan virus and Ebola virus, in which five or four affinity algorithms predicted MHC-I and MHC-II restricted epitopes, respectively, selecting epitopes with Rank ≤ 2% 3 or 2 times among toolkits. Strong cellular responses in enzyme-linked immunospot assays with corresponding epitopes validated the effectiveness of this strategy in infectious diseases such as viral hemorrhagic fever virus (29–31).

**Table 1:** The various tools involved in the article and their functions, websites.

function	platform	site
MHC-I restricted epitopes analysis	IEDB	https://www.iedb.org/
	NetMHCpan 4.1	https://services.healthtech.dtu.dk/service.php.NetMHCpan-4.1
	SYFPEITHI	http://www.syfpeithi.de/bin/MHCServer.dll/EpitopePrediction.htm
	Rankpep	http://imed.med.ucm.es/Tools/rankpep.html
	SMMPMBEC	https://github.com/ykimbiology/smmpmbec
MHC-II restricted epitopes analysis	IEDB	https://www.iedb.org/
	NetMHCIIpan3.2	https://services.healthtech.dtu.dk/service.php.NetMHCIIpan-3.2
	SYFPEITHI	http://www.syfpeithi.de/bin/MHCServer.dll/EpitopePrediction.html
	Rankpep	http://imed.med.ucm.es/Tools/rankpep.html
B-cell epitopes analysis	BepiPred-2.0	https://services.healthtech.dtu.dk/service.php.BepiPred-2.0
	IEDB B-cell epitope analysis^1^	http://tools.iedb.org/bcell/
Immunogenicity Analysis	VaxiJen v2.0	http://www.ddg-pharmfac.net/vaxijen/VaxiJen/VaxiJen.html
	IEDB Immunogenicity Analysis	http://tools.iedb.org/immunogenicity/
Conservancy Analysis	IEDB conservancy Analysis	http://tools.iedb.org/conservancy/
	Blastp	https://blast.ncbi.nlm.nih.gov/Blast.cgi
Molecular docking of 9-mer peptide epitopes	HPEPDOCK 2.0	http://huanglab.phys.hust.edu.cn/hpepdock/
Molecular docking of 15-mer peptide epitopes	EpiDOCK server	http://www.ddg-pharmfac.net/epidock/EpiDockPage.html
Molecular Structure Model Query	RCSB PDB	https://www.rcsb.org/
Tertiary structure predict	I-TASSER	https://zhanglab.ccmb.med.umich.edu/I-TASSER/
Tertiry structures refinement	GalaxyRefine server	http://galaxy.seoklab.org/cgi-bin/submit.cgi?type=REFINE
Allergen analysis	AlgPred 2.0	https://webs.iiitd.edu.in/raghava/algpred2/
Toxicity analysis	ToxinPred2	https://webs.iiitd.edu.in/raghava/toxinpred2/index.html

^1^IEDB B-cell epitope analysis tool includes six methods: Bepipred Linear Epitope Prediction, Parker Hydrophilicity Prediction, Kolaskar & Tongaonkar Antigenicity, Karplus & Schulz FlexTurn Prediction, Emini Surface Accessibility Prediction, and Chou & Fasman Beta-ibility Prediction.

### 1.2 “Integration”: Linking multiple assays and testifying each other

Most studies on bioinformatics exploration of peptide-based therapeutics lacked validation of relevant results (32–34). This may be related to the time- and resource-consuming experimental conditions. For example, considering the risk of Ebola virus and the harsh nature of the required test conditions, Alizadeh et al. resigned the evaluation after designing a multiepitope vaccine against Ebola virus (35). However, we found a connection between different types of epitope analysis, so the results of multiple analyses can be compared to validate the results at the bioinformatics level.

Take an example of the depth implication in affinity analysis and molecular docking (8). Peptide-MHC interactions are crucial in immune functions. Therefore, determining the structure of Peptide-MHC complexes is important for understanding the molecular mechanism of related biological processes and developing peptide-based immunotherapy. Typically, the RCSB PDB (https://www.rcsb.org/) is used to obtain models of MHC molecules and then HEPEDOCK 2.0 is used to predict the possibility of MHC-peptide complexes and provide multiple docking models (36). EpiDOCK toolkit can be also used in MHC-II-peptide docking and predicting binding energy (37). Binding affinity and molecular docking share a similar connotation, indicating the binding ability of MHC molecules and epitopes (38, 39). Therefore, the results of both analyses can mutually validate each other *in silico*. However, if there is a gap in the above comparison, it may be derived from the defects in algorithms’ intrinsic variety. More importantly, by simulating the docking between epitopes and MHC, the location and tightness of the docking can be directly figured out, so that the affinity data can be more intuitively reflected.

### 1.3 “Integration”: unveiling principles in comparative immunology, intriguingly

Most algorithms give only a large number of “cold” numbers in processing antigens. How to convert these numbers into meaningful models or illustrations related to the body’s immunologic nature is an aporia faced by every bioinformatics researcher. Here are three approaches to delineate the above issues.

A large amount of data will be generated in the affinity analysis of multiepitopes together with multi-MHC genotypes. It would not be state-of-art to present the whole things directly in the main text. By looking at these data using a holistic view, that is, using a heatmap form, which can show three dimensions: the epitopes, MHC genotypes and affinity ranks (40, 41). It is intuitive to exhibit the epitopes that are dominant in MHC genotypes. At the same time, bihierarchical clustering would reveal the cross-reactivity between different genotypes based on affinity analysis data (42, 43). This intuitively reflects genotype proximity according to pathogen specificity, further understanding of susceptibility and resistance against pathogens in different races, geographies, populations, and even species. Using these forms to present data not only makes the expression more intuitive and concise but also fully interprets the deep-seated meaning of the data.

Alignment of variants has been implicated in many studies but is limited to the step of multiple sequence alignment, finding mutation sites, and exploring evolutionary clades (42, 44, 45). On this basis, another direction — further research on the impact of mutation sites — should also be taken into consideration. That is, the mutation site is brought into the original sequence to explore the impact of the mutation site on the affinity and immunogenicity of the specific epitopes (29, 31). This analysis of the mutated sequences *in silico* predicts the approximate direction of evolutionary impacts on immunogenetic variation and deepens the understanding of pathogen conservation.

Epitope studies always aim to design drugs or vaccines for clinical use. Therefore, building of molecular model will be a complex and important work. At present, there are two channels to obtain molecular structure models: one is based on the existing RCSB PDB database to retrieve the discovered molecular structures, and the other is *ab initio* prediction method based on molecular dynamic (MD). That is, 3D atomic models were building from different stringing arrangements and iterative structural assembly simulations according to amino acid sequences, and then the models are optimized by MD. MD is a computer simulation method which is employed in various engineering and science disciplines to calculate motion and equilibrium of each individual atom or molecule. It can be used to explore conformational space, and is often the method of choice for large molecules such as proteins. Recently, due to the continuous development of MD, molecular models and docking are closer to the real response (46). Here, we recommend a method, the integration of I-TASSER and GalaxyRefine server. I-TASSER integrates inter-residue contact maps from deep neural-network learning with the cutting-edge fragment assembly simulations (47). GalaxyRefine performs repeated structure perturbation and subsequent overall structural relaxation by molecular dynamics simulation (48). The integration of I-TASSER and GalaxyRefine server allows the molecular structure to be rebuilt and optimized, providing an overall intuitive molecular structure (8, 49).

### 1.4 “Integration”: Adjusting cogitation according to antigenic characteristics and contradiction

In recent years, bioinformatics has been widely used on epitope studies of pathogenic microorganisms. However, *in silico* analysis is not static and needs to be adjusted according to the characteristics of antigens and the purposes of the research. The contradictions investigated in tumor immunity and autoimmune diseases are distinguishing. Also, the “elegance” of integration should be noted.

Tumor antigens are usually cancer-specific peptides (neoepitopes) (50)generated by somatic mutations or genomic aberrations, posttranslational modifications (PTMs) (51), and translation from noncoding regions (52). The aim of tumor immunity is to circumvent immune suppressive evasion mechanisms used by cancer cells through modulation of T cell responses (2). Therefore, *in silico* analysis of tumor antigens mainly focuses on interrogating cytotoxic T (CTL) cell epitopes and promoting cytotoxicity (53). On the other hand, autoimmune diseases arise from immune responses to self-antigens and reflect a breakdown in immunological tolerance (54). Most classical autoimmune diseases have associations with genes in the MHC-II region (55), leading to the production of autoantibodies (56) or the activation of CTL cells (57) to autoantigens. Therefore, pathognomonic autoimmune diseases need to be specifically designed according to the disease mechanism and site of immunoreaction.

T-cell (CD8 + and CD4 + T-cell) epitopes and B-cell epitopes are the material basis of adaptive immune responses as allure to most of studies. However, few has addressed the association among them so far. It is well known that CD4+ T cells, as helper cells, promote both killer T cells and B cells and then strengthen humoral and cellular immunity (58). Obeying this immune principle, following routine can be considered: epitopes should be validated, and the links between genuine MHC-I and MHC-II dominance, perhaps MHC-II and B-cell antigenic determinants can be found to mine the substantial bases of cellular and humoral immunity.

**Figure 1:**
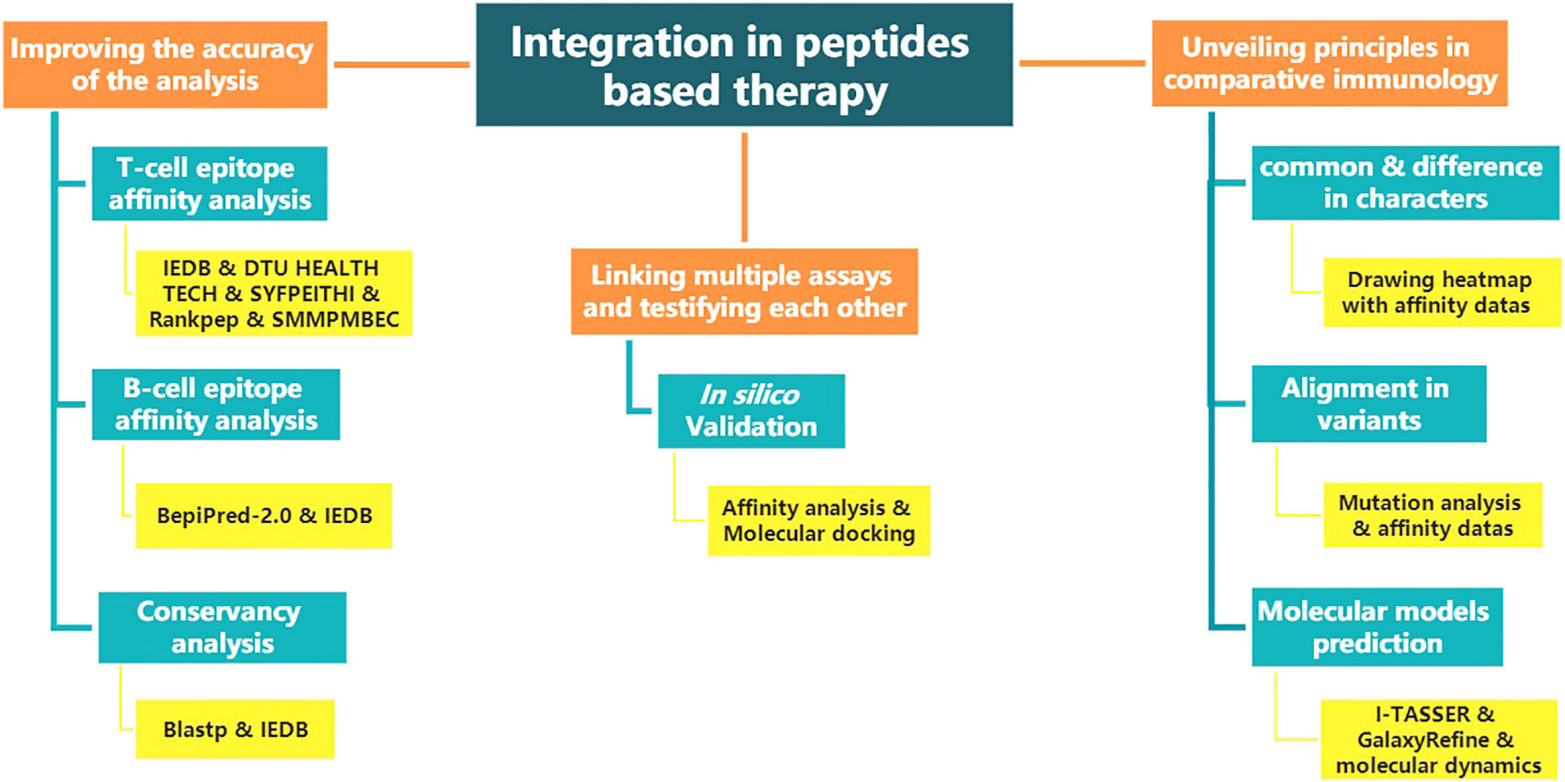
The structure diagram of recommendatory integration strategy. The orange is the purpose, blue is the method and yellow is the tool involved.

The above renders multiple “integration” ideas. **Figure 1** shows the structure diagram of recommendatory integration strategy. However, attentions should be paid in the practice: an epitope could be jointly selected with affinity, immunogenicity, conservation, toxicity, allergen analysis, etc. It would be interesting but challenging to figure out what impacts the different orders will have on the results and what particular connotations the epitopes will be endued with. Additionally, for each operation, the results will be tested once. It is intractable to face false positives, false negatives or overfitting of the integrated results due to multiple testing. Finally, toolkits of the algorithms and datasets do not update in a timely manner, which directly affects the tool selection, priority of use, and settings of various parameters.

## 2 Discussion

The evolution of epitope-based vaccines is one of the most promising developments arising from bioinformatics-based research (8), and the development of bioinformatics has also made pathogen epitope exploration convenient and cost-effective (11). We put forward a point of view, “Integration”, aiming to improve the accuracy and fit-in the body’s immune response. It says the opinion that integration manners are advances in bioinformatics rather than just a single algorithmic innovation. The acquisition of analytical results is by no means the terminus of epitopes studies. How to apply the toolkits *in silico* under the precondition for immunology principles will be critical issues to be addressed. In this opinion, the “integration” tenet was first put forward, dedicated to compensating for inherent deficiencies in current algorithms and simulating the realistic immune response model. It provided a novel train of thought for peptide-based immunotherapy *in silico* against infection, neoplasms and autoimmune diseases, and would by all odds promote the development and progress of the bioinformatics discipline.

## Author contributions

Conceptualization: DJ, BS, and KY. Methodology: DJ, BS, and JZ. Formal analysis: SB and DJ. Investigation: BS, DJ, and YoW. Resources, DJ, and KY. Writing—original draft preparation: BS and JZ. Writing—review and editing: DJ, JZ, ZL, MX, and YoW. Peer discussion: YoW, CL, LC, and YuW. Visualization, BS, JZ, ZL, and MX. Supervision, DJ and KY. Project administration, DJ and KY. Funding acquisition: DJ and KY. All authors contributed to the article and approved the submitted version.
