# Corrigendum: Integration: gospel for immune bioinformatician on epitope-based therapy

**DOI:** 10.3389/fimmu.2023.1214876

**Published:** 2023-05-24

**Authors:** Baozeng Sun, Junqi Zhang, Zhikui Li, Mingyang Xie, Cheng Luo, Yongkai Wang, Longyu Chen, Yueyue Wang, Dongbo Jiang, Kun Yang

**Affiliations:** ^1^ Department of Immunology, Basic Medicine School, Air-Force Medical University (the Fourth Military Medical University), Xi’an, Shaanxi, China; ^2^ The Key Laboratory of Bio-hazard Damage and Prevention Medicine, Basic Medicine School, Air-Force Medical University (the Fourth Military Medical University), Xi’an, Shaanxi, China; ^3^ Department of Microbiology, Basic Medicine School, Air-Force Medical University (the Fourth Military Medical University), Xi’an, Shaanxi, China; ^4^ Department of Rheumatology, Tangdu Hospital, Air-Force Medical University (the Fourth Military Medical University), Xi’an, Shaanxi, China

**Keywords:** integration, epitope, immunotherapy, *in silico*, bioinformatics, immune response

In the published article, there were two errors in the Graphical Abstract as published. In the top right-hand corner of the image, under the “CD8 + T cell” section, “CD4” should read “CD8” and on the left-hand side of the image, “Conversation” should read “Conservation”. The corrected Graphical Abstract appears below.

**Graphical Abstract:**
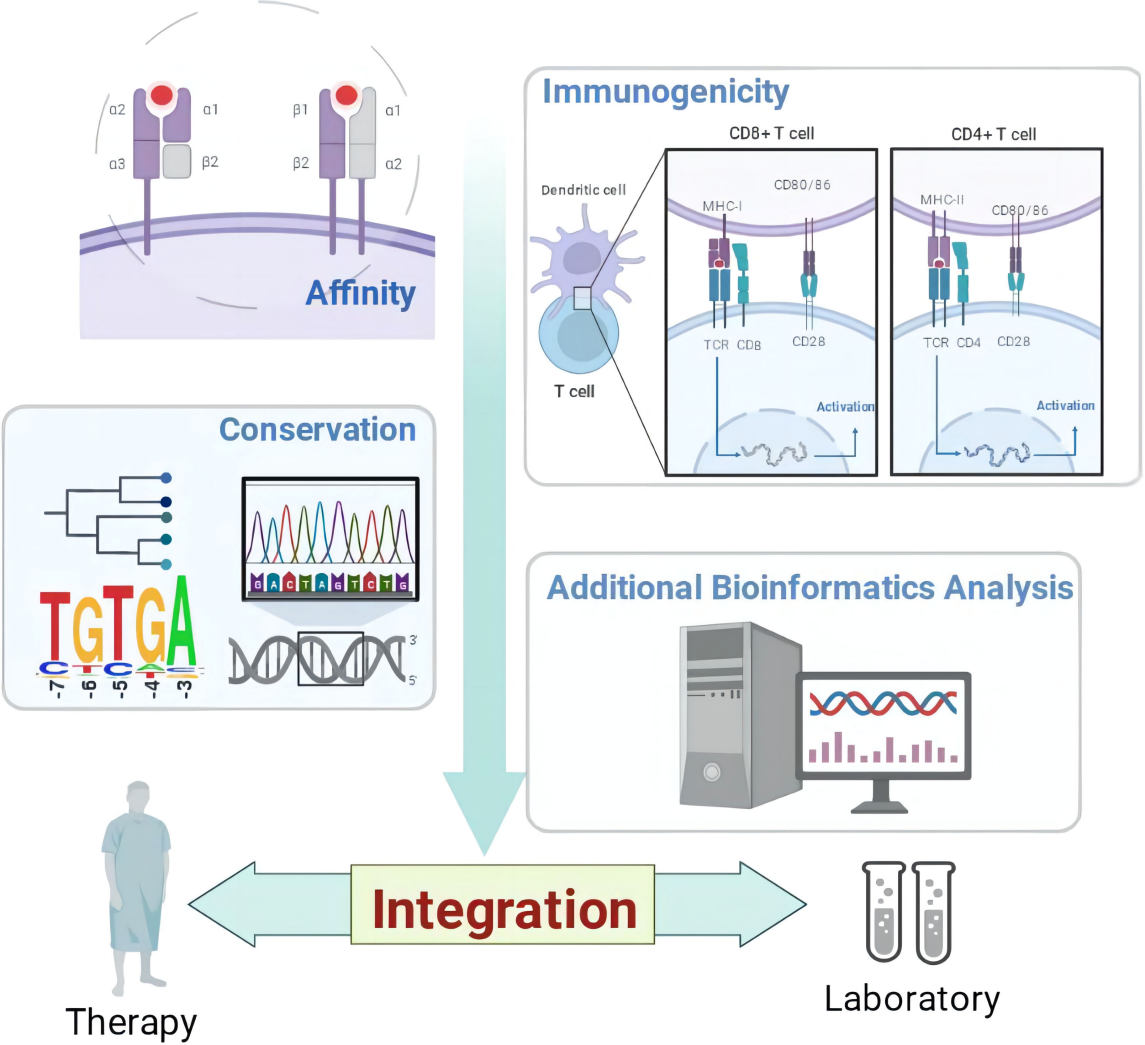


The authors apologize for this error and state that this does not change the scientific conclusions of the article in any way. The original article has been updated.

